# A critical and Integrated View of the Yeast Interactome

**DOI:** 10.1002/cfg.412

**Published:** 2004-07

**Authors:** Michael Cornell, Norman W. Paton, Stephen G. Oliver

**Affiliations:** 1 Department of Computer Science University of Manchester Kilburn Building Oxford Road Manchester M13 9PL UK; 2 School of Biological Sciences University of Manchester The Michael Smith Building Oxford Road Manchester M13 9PT UK

## Abstract

Global studies of protein–protein interactions are crucial to both elucidating gene function and producing an integrated view of the workings of living cells. High-throughput studies of the yeast interactome have been performed using both genetic and biochemical screens. Despite their size, the overlap between these experimental datasets is very limited. This could be due to each approach sampling only a small fraction of the total interactome. Alternatively, a large proportion of the data from these screens may represent false-positive interactions. We have used the Genome Information Management System (GIMS) to integrate interactome datasets with transcriptome and protein annotation data and have found significant evidence that the proportion of false-positive results is high. Not all high-throughput datasets are similarly contaminated, and the tandem affinity purification (TAP) approach appears to yield a high proportion of reliable interactions for which corroborating evidence is available. From our integrative analyses, we have generated a set of verified interactome data for yeast.


**Comparative and Functional Genomics**
*Comp Funct Genom* 2004; **5**: 382–402.
Published online in Wiley InterScience (www.interscience.wiley.com). **DOI:** 10.1002/cfg.412

**Research Article**

### A critical and integrated view of the yeast interactome

Michael Cornell[1,2]*, Norman W. Paton[1] and Stephen G. Oliver[2]

[1] *Department of Computer Science, University of Manchester, Kilburn Building, Oxford Road, Manchester M13 9PL, UK*
[2] *School of Biological Sciences, University of Manchester, The Michael Smith Building, Oxford Road, Manchester M13 9PT, UK*

**Correspondence to: Dr Michael Cornell, Dept of Computer Science, Kilburn Building, Oxford Road, Manchester M13 9PL, UK. E-mail: mcornell@cs.man.ac.uk*

### Abstract

**Global studies of protein–protein interactions are crucial to both elucidating gene function and producing an integrated view of the workings of living cells. High-throughput studies of the yeast interactome have been performed using both genetic and biochemical screens. Despite their size, the overlap between these experimental datasets is very limited. This could be due to each approach sampling only a small fraction of the total interactome. Alternatively, a large proportion of the data from these screens may represent false-positive interactions. We have used the Genome Information Management System (GIMS) to integrate interactome datasets with transcriptome and protein annotation data and have found significant evidence that the proportion of false-positive results is high. Not all high-throughput datasets are similarly contaminated, and the tandem affinity purification (TAP) approach appears to yield a high proportion of reliable interactions for which corroborating evidence is available. From our integrative analyses, we have generated a set of verified interactome data for yeast.** **Copyright © 2004 John Wiley & Sons, Ltd.**

Received: 10 October 2003
Revised: 23 April 2004
Accepted: 14 May 2004

**Keywords:** ***Saccharomyces cerevisiae*; protein interactions; data integration**

**Supplementary material for this article can be found at http://www.interscience.wiley.com/jpages/1531-6912/suppmat**

### Introduction

The function of most proteins is dependent on their interaction with other molecules, including other proteins. Therefore, in order to gain the global appreciation of protein function demanded by functional genomics, it is essential to identify the totality of protein–protein interactions, the 'interactome' (Rain *et al*., 2000).

The development of high-throughput techniques to produce large functional datasets, in a paradigm termed 'system-driven' biology (Blackstock and Mann, 2000), is enabling experimentalists to amass large amounts of interactome data (Uetz *et al*., 2000; Ito *et al*., 2001; Gavin *et al*., 2002; Ho *et al*., 2002). The 'system-driven' approach has the advantage that the data produced are much more comprehensive and less likely to be biased towards the specific interests of an individual investigator than those obtained by more traditional methods. However, these high-throughput techniques can generate a significant proportion of false-positive identifications and the size of the datasets precludes follow-up experiments to eliminate them. In order to maximize the usefulness of these functional datasets, it is important that means be developed to allow the assessment of data quality and the reduction of contaminating false-positive results.

One way to evaluate interactome data is by cross-validating independent protein-interaction screens and interrelating interactome data with additional information from other sources (von Mering *et al*., 2002). We have developed the Genome Information Management System (GIMS) (Cornell *et al*.,

Copyright © 2004 John Wiley & Sons, Ltd.

2003), an object-oriented database that integrates a model of the *S. cerevisiae* genome with transcriptome, interactome and other functional data (Paton *et al*., 2000). GIMS has been used to analyse four recently published interactome datasets, two obtained by yeast two-hybrid (Y2H) screens (Uetz *et al*., 2000; Ito *et al*., 2001) and two by protein complex purification (Ho *et al*., 2002; Gavin *et al*., 2002). This has enabled us to estimate the extent to which these datasets are contaminated by false-positive results (i.e. interactions that are identified in experiments but do not represent genuine interactions in the cell). GIMS has been used to identify those interactions which are supported by multiple datasets, rather than being peculiar to a particular screen. Interactions have been compared using correlation of mRNA expression profiles across a set of 300 microarray experiments (Hughes *et al*., 2000), and comparison of protein annotation for cellular localization and function from GO (Ashburner *et al*., 2000) and MIPS (Mewes *et al*., 1997). Our hypothesis is that interacting proteins may be expected to share annotations and have similar expression profiles. This has previously been shown for a set of complexes identified in traditional studies and listed in MIPS (Jansen *et al*., 2002). Our results indicate that the lack of overlap between interactome datasets is due more to the reporting of false-positives than to the small proportion of all interactions sampled by a specific screen. We find that some experimental methods appear to produce fewer false-positive interactions than others. The affinity-purified complexes generated by the TAP method (Gavin *et al*., 2002) were found to be more reliable than those generated by the HMS–PCI technique (Ho *et al*., 2002), while the Y2H interactions reported by Uetz *et al*. (2000) appear more reliable than those reported by Ito *et al*. (2001). Our analyses were performed using the GIMS database, which can be accessed via a Java application from **http://www.cs.man.ac.uk/img/gims/software.html**

### Materials and methods

### The genome information management system

GIMS is an object database incorporating a model of the *Saccharomyces cerevisiae* genome plus a range of functional data. Full descriptions on the downloading and use of the GIMS application and the database itself are available (Paton *et al*., 2000; Cornell *et al*., 2003).

### Protein interaction data

Published yeast two-hybrid (Ito *et al*., 2001; Uetz *et al*., 2000) and affinity-purified complexes (Gavin *et al*., 2002; Ho *et al*., 2002) were stored in the GIMS database. Yeast two-hybrid data are stored in the database using two classes. *ProteinProteinInteraction* objects record that two proteins interact. These objects contain links to one or more *InteractionExperimentResult* objects, which record the bait protein, the investigators name and the number of times this interaction was recorded in the screen, if this information has been provided. Ito Y2H interactions can be subdivided into two groups: the core data (interactions identified more than three times in the screen) and the reminder.

Protein complex data is stored in the classes *MipsDefinedComplex* and *AffinityPurifiedComplex*, both subclasses of *ProteinComplex*. A fragment of the GIMS schema showing classes used to model protein interaction data is shown in the Supplementary Information (see **http:www.interscience.wiley.com/jpages/1531-6912/suppmat**).

There are a few instances in which interactions could not be included in GIMS because they involve genes that are no longer thought to be real and are now excluded from MIPS. In addition, we found instances where affinity-purified complexes were duplicated in published datasets, or where a protein was included in a complex more than once. Interactions excluded from our analysis and protein duplications are listed in the Supplementary Information.

### Protein annotation

GO terms (Biological Process, version 2.1177, and Cellular Component, version 2.454) and association of GO terms with *S. cerevisiae* gene products (version 1.834; date, 3 April 2004) were downloaded from the Gene Ontology website **http://www.geneontology.org/index.shtml**.

MIPS annotations for subcellular localization (dated 21 March 2003), and protein classes (5 October 2001) were downloaded from the CYGD ftp site, **http://mips.gsf.de/desc/yeast**.

Copyright © 2004 John Wiley & Sons, Ltd. 

### Comparison of annotation for pairs of proteins

For the purposes of our analyses, we consider a protein pair to be either a pair of proteins that directly interact, or which are associated together in the same complex. For proteins pairs where both had MIPS or GO annotation, the annotations were compared. The GO terms GO:0008372 (cellular component unknown) and GO:0000004 (biological process unknown) and the MIPS subcellular localization term 799 (other subcellular localization) and functional categories (98 classification not yet clear-cut) and 99 (unclassified proteins) were not used for comparison.

If the two proteins share an annotation term, the protein pair was scored as a 'match'. If none of the terms is shared, this was scored as a 'no-match'. For example, a yeast two-hybrid interaction has been identified between Ypl031p and Ydl127p (Uetz *et al.*, 2000). Ypl031p is associated with four biological process GO terms: GO:0005977 (glycogen metabolism); GO:0006796 (phosphate metabolism); GO:0006468 (protein amino acid phosphorylation); GO:0007049 (cell cycle). Ypl031p is also associated with GO:0007049, therefore this yeast two-hybrid interaction is scored as a match. For a set of protein pairs, the total match : no-match ratio was calculated. The match and no-match scores used to calculate these ratios are provided in the Supplementary Information.

When analysing protein pairs in affinity purification datasets, the same pairs of proteins can occur in multiple analyses. In order to ensure our analyses are not biased towards frequently occurring pairs, each pair of proteins is only considered once. However, the frequent occurrence of protein pairs is of interest and we have performed separate analyses of frequently occurring and single-occurrence pairs.

### Assessment of GO annotations for protein comparisons

Associations of GO terms with gene products have evidence fields describing the basis of the association. There is a loose hierarchy of reliability of evidence types, with Traceable Author Statement (TAS) and Inferred from Direct Annotation (IDA) as the as the most reliable, and Inferred by Electronic Annotation (IEA) as the least (for more details, see **http://www.geneontology.org/GO.evidence.html**). In order to confirm that differences between interaction datasets were not due to the reliability of the annotation, we compared the evidence codes for association of GO terms with proteins in each dataset. For each dataset, there are no IEA-based associations and the percentage of associations with TAS or IDA evidence codes are similar (approximately 60%). For further details, see the Supplementary Information.

### Use of the GO DAG for annotation comparisons

In addition to directly comparing associated GO terms, we have investigated comparing annotations at different levels of the GO directed acyclic graph (DAG). There are clearly issues to be considered with these types of analyses. Should 'is a' and 'part of' parent–child relationships be considered equal, and how far should the DAG be navigated for a comparison? In order to investigate the effect of including parent terms in the analyses, we repeated the analyses of annotation of proteins in yeast two-hybrid interactions. As well as comparing associated GO terms, we compared the parents of these terms, provided that the parent terms were not GO:0005575 (cellular component) or GO:0008150 (biological process). The results of this comparison (see Supplementary Information) show that although match : no-match ratios increased when parent terms were included, the overall differences between interaction datasets were unchanged.

### Assessment of transient interactions in datasets

Proteins likely to be associated with transient interactions were copied from the CYGD website (25 March 2003). The 462 proteins were associated with MIPS functional classes 41.11.21 (metalloproteases), 51 (cyclins), 115 (histone acetyltransferases and histone deacetyltransferases), 151 (proteases), 161 (protein kinases), 181 (protein phosphatases), 191 (ubiquitin-system proteins) and 201 (transcription factors). Sets of protein pairs were assessed to see if either protein was in the set of transient interacting proteins. If either or both were, the interaction was classified as transient.

### Analysis of microarray expression data

Microarray data (Hughes *et al.*, 2000) were downloaded from the Rosetta Inpharmatics website **http://www.rii.com**. Pearson correlation coefficients of expression profiles for pairs of genes were

Copyright © 2004 John Wiley & Sons, Ltd. 

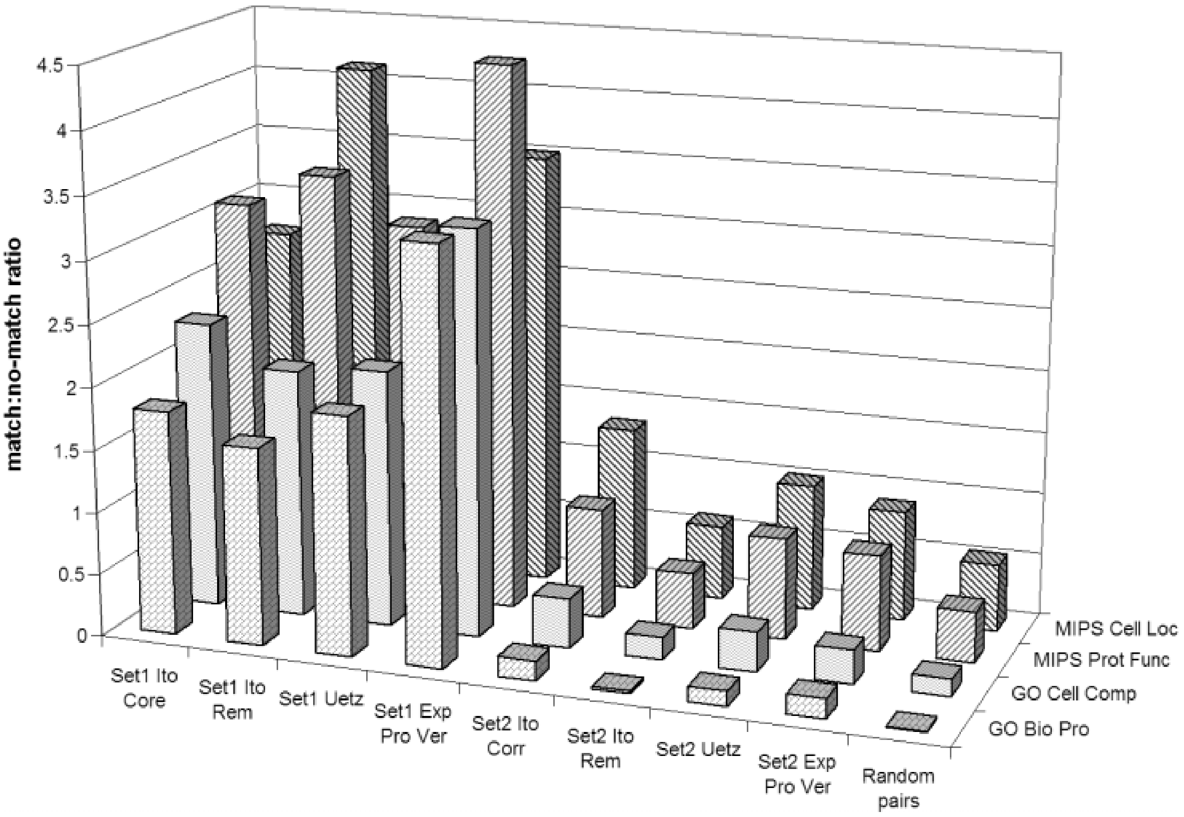

**Figure 1.** Comparison of annotation 'match : no-match' ratios for Set1 and Set2 yeast two-hybrid interactions. Key: Set1 Ito Core, interactions in the Ito core set which occur in Set1; Set1 Ito Rem, interactions in the Ito remainder set which occur in Set1; Set1 Uetz, interactions in the Uetz data set which occur in Set1; Set1 Exp Pro Ver, interactions in the Ito core set which occur in an expression profile verified set (Kemmeren *et al.*, 2002); Set2 Ito Core, interactions in the Ito core set which occur in Set2; Set2 Ito Rem, interactions in the Ito remainder set which occur in Set2; Set2 Uetz, interactions in the Uetz data set which occur in Set2; Set2 Exp Pro Ver, interactions in an expression profile verified set (Kemmeren *et al.*, 2002) which occur in Set2; random pairs, a set of 4010 pairs of randomly chosen proteins calculated using $\log_e$ ratio values. The relative frequency distributions of the correlation coefficients were calculated for protein interaction datasets. Where statistical differences between sets of protein pairs are reported, they were determined by calculating the probability that the distributions were the same using two-tailed Student's $t$-tests (two sample, unequal variance).

### Results

### Assessment of overlap between interaction datasets

In order to gain an insight into the degree of overlap between datasets we have asked the following questions:

- *Question 1*: *Ninety-four bait proteins are common to both the TAP and HMS-PCI datasets. What is the overlap between the sets of proteins they identify?* Proteins identified by these baits were compared. In some cases, there is a good overlap between the datasets. For example, eight of the ten proteins in the TAP complex purified using Erb1p were also identified in the HMS–PCI complex (although the HMS–PCI complex for this bait contained a total of 39 proteins). However, such cases are the exception. On average, the number of proteins common to both datasets is less than 9% of the total number of proteins in both datasets. Examples of complexes with low overlap include those purified using Pph22p as bait, which identified 16 proteins using TAP and 13 using HMS–PCI.

Copyright © 2004 John Wiley & Sons, Ltd. 

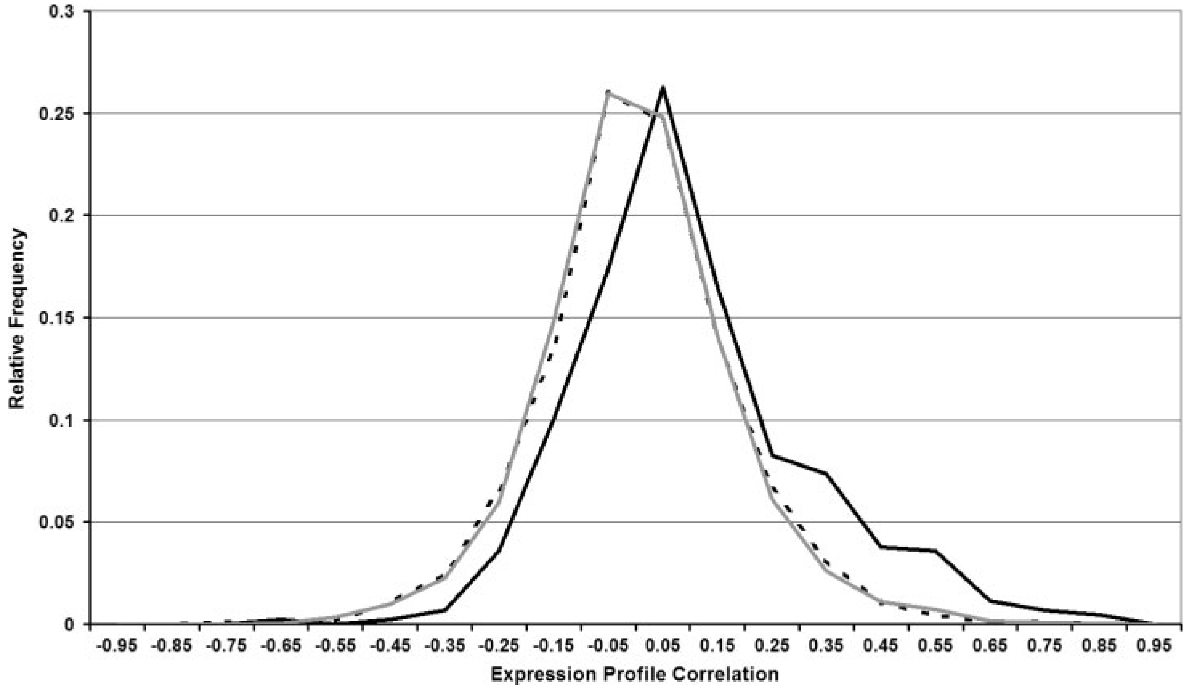

**Figure 2.** Frequency distribution of expression profile correlations for Set1 and Set2 yeast two-hybrid interactions and randomly chosen pairs of proteins. Key: black solid line, Set1 interactions; black dashed line, Set2 interactions; grey solid line, random pairs

None of these proteins is common to both. Employment of Yju2p as a bait identified 15 proteins using TAP and 15 using HMS–PCI. Only one protein (Prp19p) is common to both. In addition, there can be considerable disparity between the size of complexes generated by TAP and HMS–PCI. Complexes generated using baits Pwp2p and Kap104p contain 54 and four proteins, respectively, using TAP, compared to seven and 36 using HMS–PCI.

- *Question 2*: *In an affinity purification screen, bait A identifies a set of proteins including B. If B is used as a bait, does it identify A?* TAP is more successful at identifying reverse interactions than HMS–PCI. In 39% of instances where B is used as a bait, it identifies A, compared to 19% for HMS–PCI.
- *Question 3*: *A protein interaction is identified by Y2H. If the same bait is used in an affinity purification will it identify the same protein?* Since Y2H screens predict a direct interaction between a bait and a prey protein, members of such interacting pairs identified by Y2H screens should also be identified in affinity-purified complexes. The largest overlap is between the TAP and the Uetz Y2H datasets, where 21% of the interactions found by Y2H are supported by affinity purification. In contrast, less than 7% of the Y2H interactions in the Ito dataset are supported by TAP. For many bait proteins, there is little overlap between the datasets. For example, 95 interactions have been reported when using Ser3p (Yer081p) as a bait (Ito *et al.*, 2001). Using the same bait for TAP purification yielded only three proteins, of which only one (Ser33p/Yil074p) was amongst those identified by Y2H. Similarly, the protein Tem1p (Yml064p) was used as a bait in four separate purification regimes using the HMS–PCI approach, thereby identifying complexes containing between three and 34 proteins. In contrast, when Tem1p was used as a Y2H bait, 24 proteins were identified (Uetz *et al.*, 2000). However, none of these proteins is identified by both methods.
- *Question 4. For two Y2H experiments involving the same bait protein, how many interactions are*

Copyright © 2004 John Wiley & Sons, Ltd.

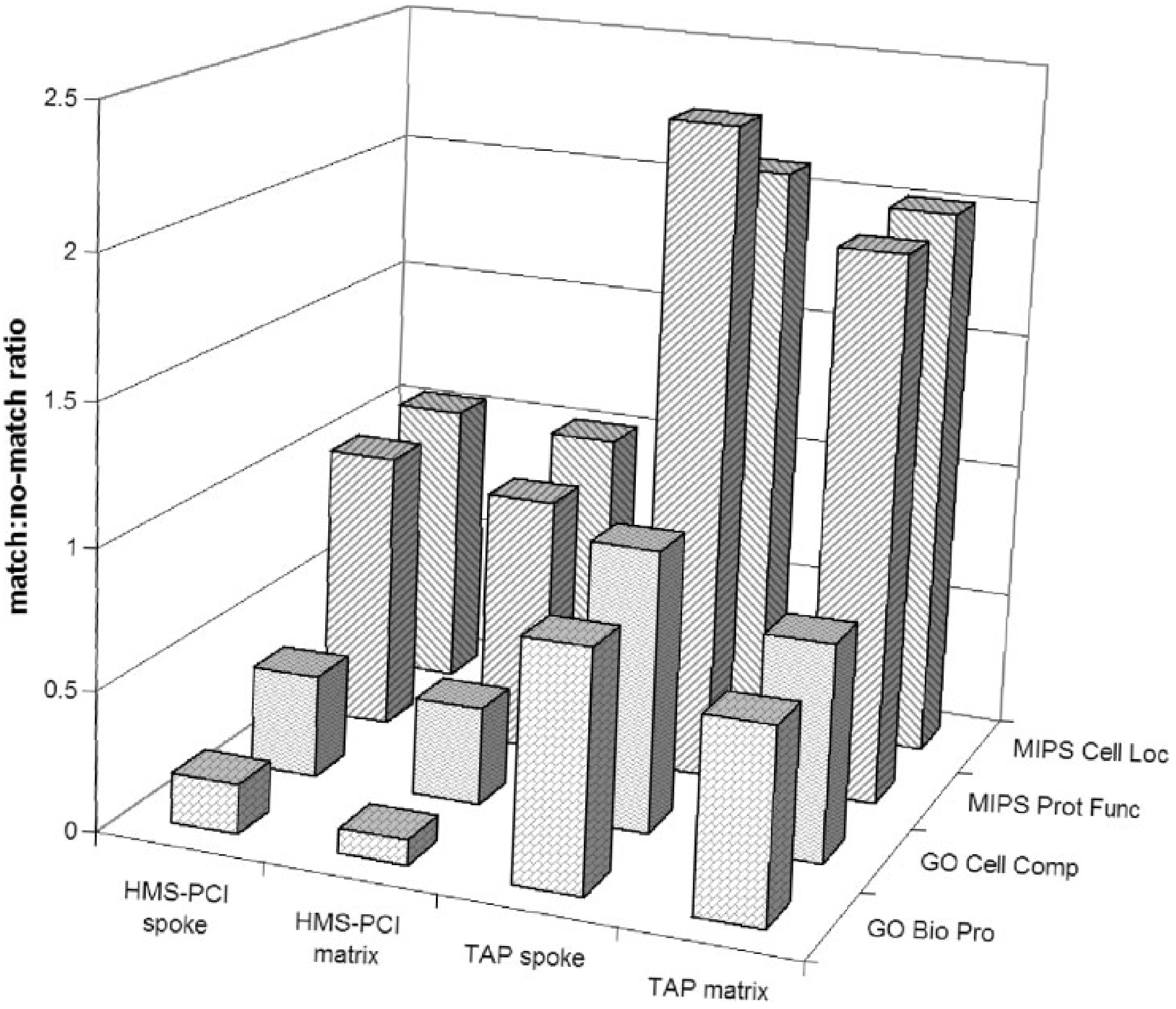

**Figure 3.** Comparison of annotation 'match : no-match' ratios for pairs of proteins in affinity-purified protein complexes generated using spoke and matrix models

*found in both the Ito and Uetz datasets?* There are 220 bait proteins in common between the two screens. For these baits, the Ito dataset contains 871 interactions, while that of Uetz contains 430; 164 interactions were common to both datasets. In some instances, there was good agreement between the two Y2H datasets. Using Ygr058p as bait, both datasets have four interactions, of which three are common to both. In comparison, using Tem1p (Yml064p), the Ito set contains 54 interacting proteins and the Uetz set 24; only six are common to both datasets.

- *Question 5. In a yeast two-hybrid screen, bait protein A identifies protein B. If B is used as a bait does it identify A?* In 106 cases, reverse interactions could be found. In 2061 instances the reverse interaction has not been identified despite the appropriate bait (i.e. protein B) being used in a given screen. In a further 2933 cases, protein B was not used as a bait in either Y2H screen.

The above analysis indicates that there is little overlap between experimental datasets. Two factors might be responsible for this. First, the large size of the yeast interactome means that any screen can only identify a fraction of all interactions (Hazbun and Fields, 2001). Alternatively, Y2H and affinity purification may both produce false-positives with the lack of overlap between datasets reflecting the relative extent of contamination.

Copyright © 2004 John Wiley & Sons, Ltd. 

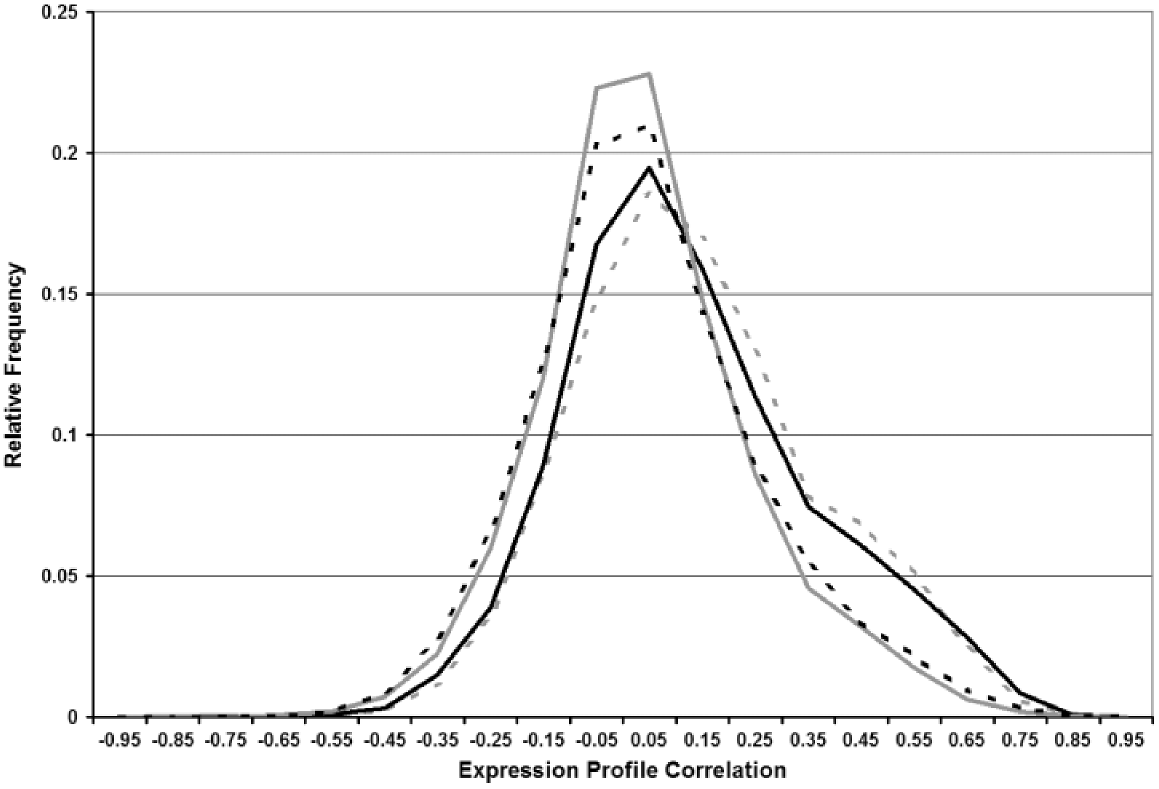

**Figure 4.** Frequency distribution of expression profile correlations for pairs of proteins in affinity-purified protein complexes generated using spoke and matrix models. Key: grey dashed line, TAP complexes, spoke pairs; black solid line, TAP complexes, matrix pairs; grey solid line, HMS–PCI complexes, spoke pairs; black dashed line, HMS–PCI complexes matrix pairs

To assess whether the lack of overlap between Y2H datasets is due to contamination, two sub-sets of Y2H protein–protein interactions were produced. Set1 contains interactions are supported by more than one dataset. This means that the interaction occurs in more than one Y2H dataset, or the reverse interaction has been identified, or the two proteins have occur in a protein complex, either as defined in the MIPS catalogues or in an affinity-purified complex. Set1 contains 466 interactions involving 598 proteins; 374 of the Set1 interactions are in the Ito dataset (243 in the core set and 131 in the remainder), while 280 are in the Uetz set. Set2 comprises the remaining 4749 interactions, which involve 3439 proteins; 496 of the proteins in Set1 interactions are also involved in Set2 interactions. In addition, 920 protein interactions verified using microarray expression data (Kemmeren *et al.*, 2002) were analysed; 202 of these interactions are in Set1, the remaining 718 in Set2. The Set1 interactions are listed in the Supplementary Information.

### Annotation comparison for protein pairs yeast two-hybrid interactions

Results for protein annotation comparisons are summarized in Figure 1. If the lack of overlap between datasets is due to the size of the interactome, rather than false-positives, the annotation comparisons should be the same for Set1 and Set2 interactions. Clearly this is not the case: Set1 interactions have much larger ‘match : no-match’ ratios (i.e. a much larger proportion of instances in which proteins predicted to interact share annotations) than the other datasets in all the comparisons made. Set1 interactions have ratios between four times (for MIPS cellular localizations) and 39 times (for GO biological processes) greater than Set2 interactions. For Set1 Ito interactions, it does not appear to matter whether they are in the core or remainder

Copyright © 2004 John Wiley & Sons, Ltd. 

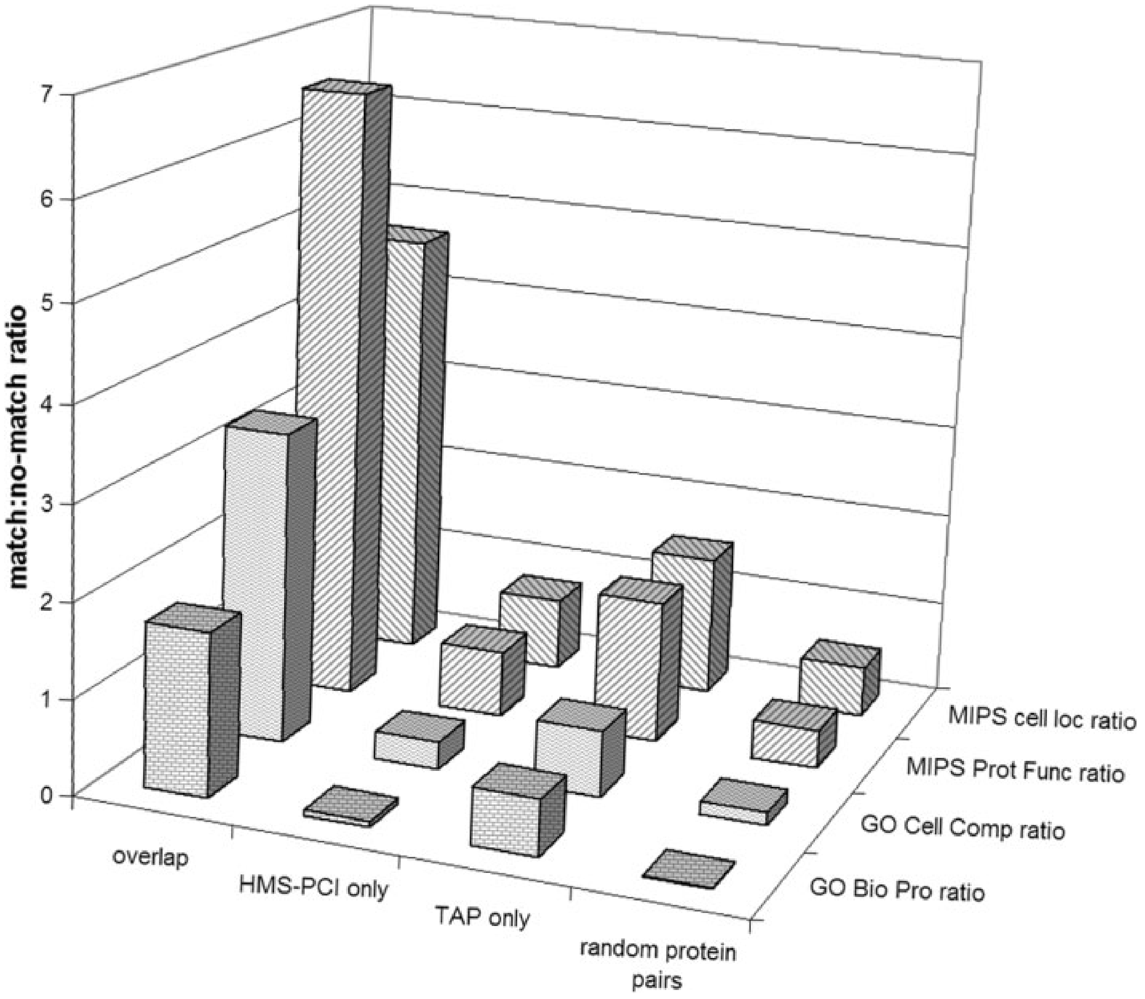

**Figure 5.** Comparison of annotation 'match : no-match' ratios for pairs of proteins in affinity-purified complexes, purified using the same bait protein by TAP and HMS-PCI. Key: overlap, pairs of proteins common to both TAP and HMS–PCI complexes; HMS-PCI only, pairs of proteins purified only by HMS–PCI; TAP only, pairs of proteins purified by TAP only; random pairs, a set of 4010 pairs of randomly chosen proteins sets. In contrast, the 'match : no-match' ratios for Set2 Ito remainder interactions are similar to those for randomly chosen pairs. The Set2 Kemmeren interactions have similar match : no-match ratios to the Set2 Ito core and Uetz interactions, much lower than those for the Kemmeren interactions in Set1.

### Expression profile correlation for protein pairs yeast two-hybrid interactions

Frequency distributions of expression profile correlations are shown in Figure 2. A previous analysis of expression profile correlation for yeast two-hybrid interactions demonstrated that they behave like random pairs of proteins (Jansen *et al.*, 2002). However, our analysis shows that, while Set2 interactions have the same distribution as random pairs ($t$-test probability $= 0.61$), Set1 interactions behave differently ($t$-test probability $= 3 \times 10^{-18}$) and tend to have greater positive correlations.

Therefore, Set1 and Set2 interactions behave very differently in comparisons of both annotation and expression profiles. This suggests that Y2H interactions identified in more than one screen are more reliable than those identified in a single screen and that the lack of overlap between datasets is largely due to the reporting of false-positives. We would expect that interactions that are identified many times in a single screen to be more reliable

Copyright © 2004 John Wiley & Sons, Ltd. 

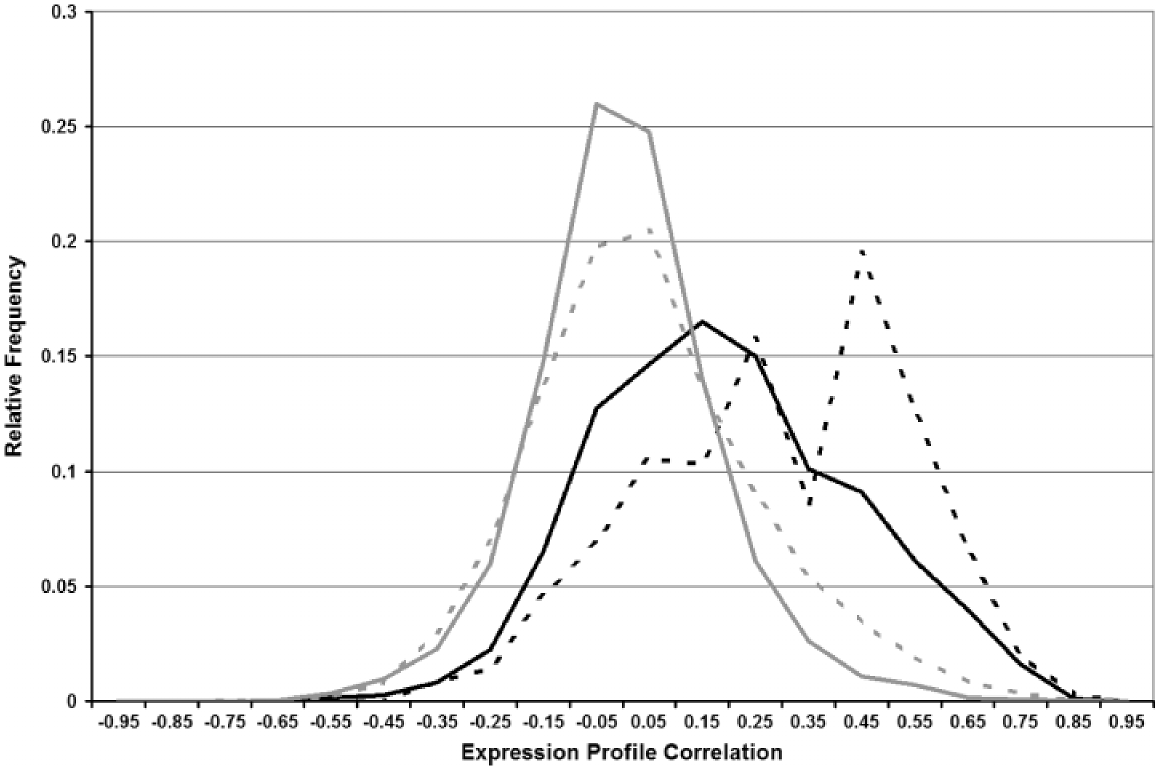

**Figure 6.** Frequency distribution of expression profile correlations for pairs of proteins in affinity-purified protein complexes, purified using the same bait protein by TAP and HMS–PCI. Key: black dashed line, overlap — pairs of proteins common to both TAP and HMS–PCI complexes; grey dashed line, HMS–PCI only — pairs of proteins purified only by HMS–PCI; black solid line, TAP only — pairs of proteins purified by TAP only; grey solid line, random pairs — a set of 4010 pairs of randomly chosen proteins

(von Mering *et al.*, 2002). However, the interaction between the Apg17p (a protein involved in autophagy) and Mrp4p (a mitochondrial ribosomal protein) was identified 52 times by Ito *et al.*, despite the fact that there is no apparent overlap between the locations or functions of these proteins. It is also clear that, while the reporting of false-positives is a serious problem, some of the interactions in Set2 are real. For example, the interaction between Srb7p and Soh1p, identified 51 times by Ito *et al.*, is supported by data on a human protein complex containing orthologues of these two proteins (Gu *et al.*, 1999).

### Analysis of affinity-purified complexes

### Comparison of spoke and matrix models for affinity-purified complexes

Two methods for modelling interactions within affinity-purified complexes have been proposed. The 'spoke' model (Bader *et al.*, 2002) only considers interactions involving the bait protein. In contrast, the 'matrix' model considers all possible pairs of proteins within the complex (von Mering *et al.*, 2002). The advantage of the spoke model is interactions between bait and identified proteins are more likely to be correct (i.e. in agreement with published literature) than interactions between identified proteins (Bader *et al.*, 2002). While this may be true, it should not be taken as meaning that the spoke model correctly models the interactions within a complex. An affinity purification experiment does not directly provide information as to the interactions within the complex. In a large affinity-purified complex, such as the Apg12-purified complex, which contains 78 identified proteins (Ho *et al.*, 2002), it seems unlikely that the bait protein could interact with all the identified proteins. In addition, the fact that interactions between bait and identified proteins are more likely to be correct than

Copyright © 2004 John Wiley & Sons, Ltd. 

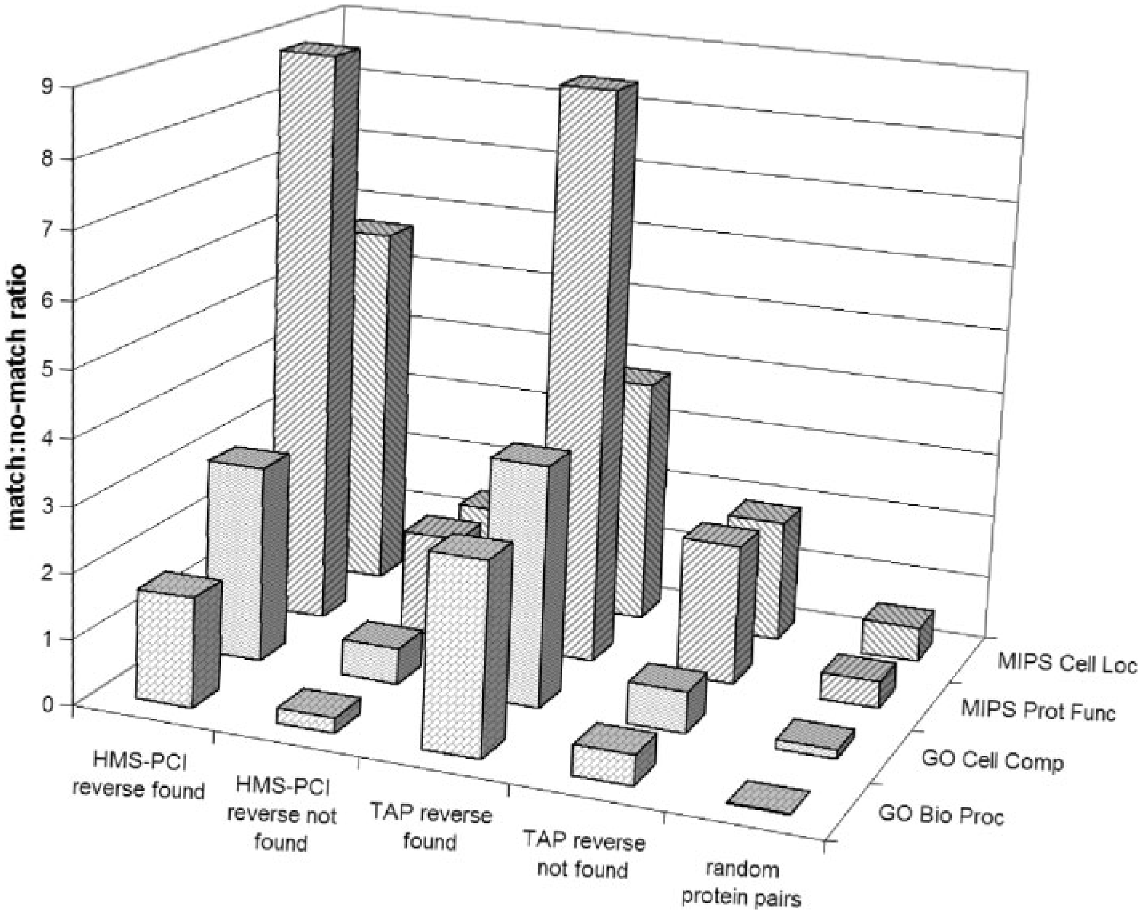

**Figure 7.** Comparison of annotation 'match : no-match' ratios for pairs of proteins in affinity-purified complexes depending on whether the reverse interaction has been identified, i.e. bait protein X has been used as a bait and purifies a complex containing Y. If Y is used as a bait, does it's complex contain X?

those between identified proteins could be a reflection on the numbers of false-positives reported among the identified proteins. Similarly, the matrix model should not be seen as representing all the interactions within a complex. Clearly, within a large complex, it is impossible for each protein to directly interact with all other proteins. Instead, it provides a mechanism for comparing all possible pairs of proteins to see if they share common features.

We compared TAP and HMS–PCI complexes using the spoke and matrix models using comparison of annotation. The spoke model generates 3163 protein pairs from TAP complexes and 3503 from HMS–PCI. The matrix model generates 17 281 protein pairs from TAP complexes and 30 672 pairs from HMS–PCI. The results, shown in Figure 3, show that differences in match : no-match ratios between the two models are far less than the differences between affinity purification methods. For all comparisons, TAP has larger match : no-match ratios than HMS–PCI. The frequency distributions for correlation of expression profiles are similar for spoke and matrix models (see Figure 4). Again, protein pairs from TAP complexes tend to have higher expression profile correlations than those from HMS–PCI complexes whichever model is chosen.

Because the choice of model does not appear to have any great impact on the results of our analyses, all further analyses of affinity-purified complexes were conducted using the matrix model. This has the additional advantage that complexes for which the bait protein has no annotation will not be excluded from the analyses as they would using the spoke model.

Copyright © 2004 John Wiley & Sons, Ltd.

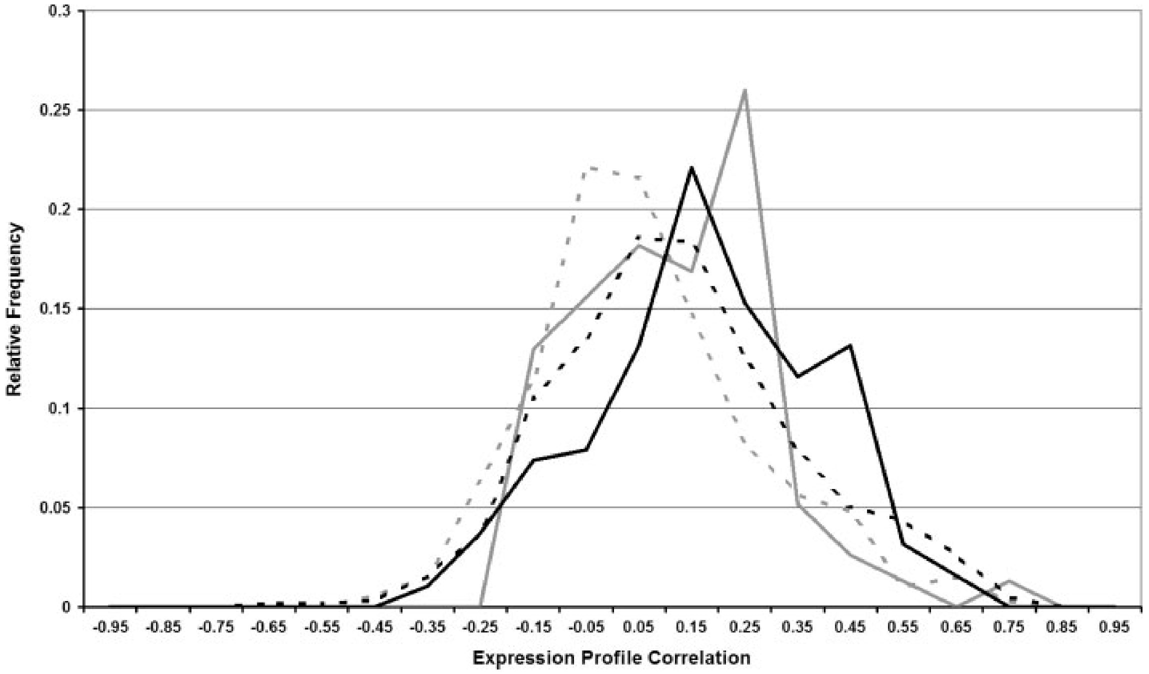

**Figure 8.** Frequency distribution of expression profile correlations for pairs of proteins in affinity-purified protein complexes, depending on whether the reverse interaction has been identified. Key: grey solid line, HMS–PCI complexes — reverse interaction found; grey dashed line, HMS–PCI complexes — reverse interaction not found; black solid line, TAP complexes — reverse found; black dashed line, TAP complexes, reverse not found

**Analysis of overlap between TAP and HMS–PCI complexes purified using the same bait**

We have shown that the overlap in the sets of identified proteins for TAP and HMS–PCI purified complexes is low (see Question 1 above). As for Y2H interactions, this could be due to either the size of the interactome or the numbers of false-positives reported. For same-bait complexes, we compared annotation and calculated expression profile correlation for pairs of proteins found by both methods and pairs found by only one method.

The result of the annotation comparisons is shown in Figure 5. Clearly, protein pairs identified by both methods have the largest match : no-match ratios, while those identified only using HMS–PCI have the smallest. Correlation of expression profiles, shown in Figure 6, gives a similar result. Proteins identified by both methods tend to have the highest correlations, while those identified by HMS–PCI only have the lowest.

**Analysis of affinity purification data for which reverse interactions are found**

We have shown that TAP is more successful at identifying reverse interactions than HMS–PCI (see Question 2 above). Do those interactions for which the reverse interaction is identified give similar match : no-match ratios as those where it is not?

The results of annotation comparisons show that they do not (see Figure 7). For each of the four comparisons, the match : no-match ratio for interactions where the reverse is found are at least twice the ratios of those where it is not. A similar result is obtained by expression profile correlations (see Figure 8), 'reverse found' pairs tend to have higher correlations than 'reverse not found' pairs. However, it is interesting to note that for 'reverse not found' TAP, protein pairs tend to have greater correlation of expression than the 'reverse found' HMS–PCI protein pairs.

Affinity purification is not reliable enough to warrant complete acceptance of the resulting data without experiments being carried out multiple times (Bader and Hogue, 2002). We would expect that, as was observed for the Y2H interaction data, identified proteins which are supported by multiple datasets are more reliable than those in a single dataset. This appears to be the case; protein pairs identified in both published screens,

Copyright © 2004 John Wiley & Sons, Ltd. 

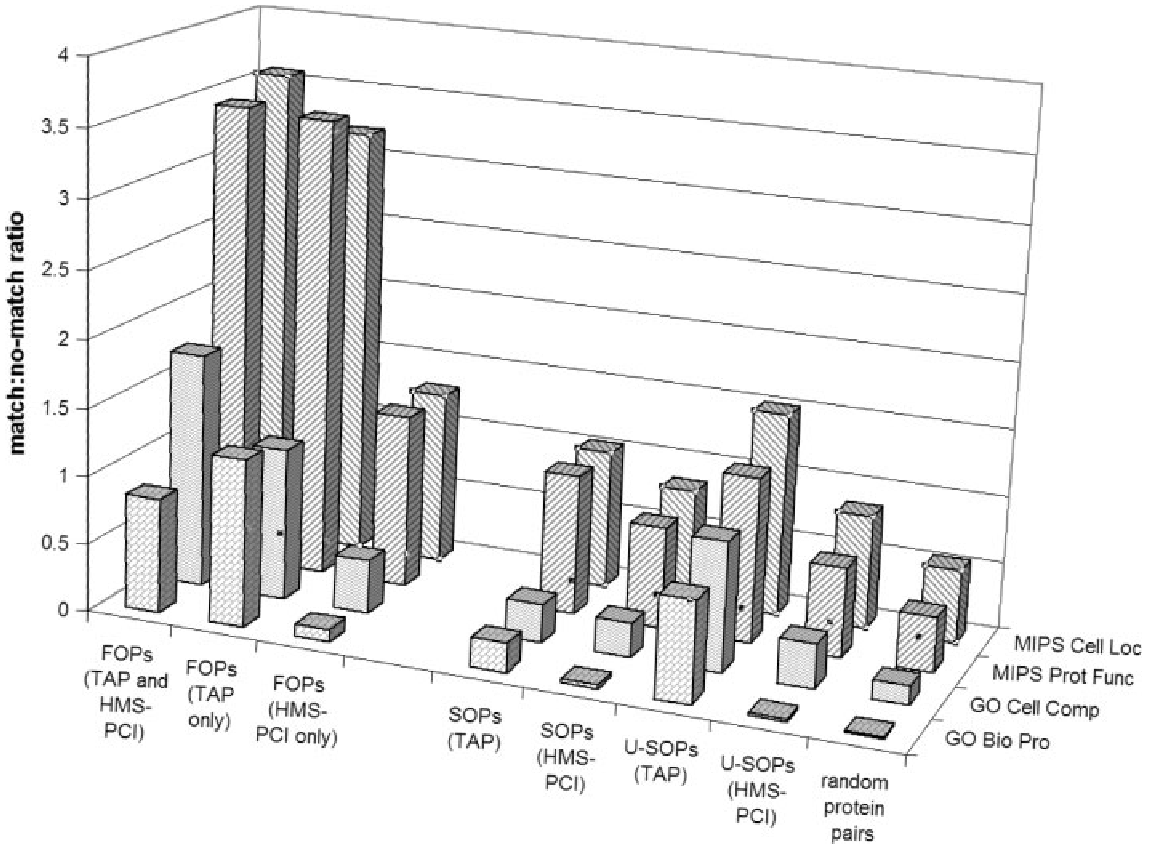

**Figure 9.** Comparison of annotation 'match : no-match' ratios for all affinity-purified complex protein pairs. Key: FOPs TAP only, frequently observed pairs which occur only in TAP-purified affinity-purified complexes; FOPs TAP and HMS-PCI, frequently observed pairs which occur in both TAP and HMS–PCI-purified affinity-purified complexes; FOPs HMS–PCI only, frequently observed pairs which occur only in HMS–PCI-purified affinity-purified complexes; U-SOPs TAP, unique single observation pairs occurring in TAP-purified affinity-purified complexes; U-SOPs HMS–PCI, unique single observation pairs occurring in HMS–PCI-purified affinity-purified complexes; SOPs TAP, non-unique single observation pairs occurring in TAP-purified affinity-purified complexes; SOPs HMS-PCI, non-unique single observation pairs occurring in HMS–PCI-purified affinity-purified complexes; random pairs, a set of 4010 pairs of randomly chosen proteins or for which a reverse interaction was observed, have higher 'match : no-match' ratio scores and expression profile correlation scores than those identified by only one. Therefore, it appears that overlapping datasets can be used to generate a set of reliable interactions.

However, lack of overlap between the HMS–PCI and TAP screens means that most of the data would be discarded. In order to overcome this problem, the occurrence of pairs of proteins within complexes was analysed. For example, the proteins Rpt4p and Rpn8p co-occur in nine affinity-purified complexes (purified by TAP using Rpn10p, Rpn12p, Rpn5p, Unp6p, Rpt2p and Rpn6p; and by HMS–PCI using Rpt3p, Arp2p and Ygl004p). Neither Rpt4p nor Rpn8p has been used as a bait protein and none of the proteins used to identify them has been used more than once. Nevertheless, the fact that they co-occur in complexes could be an indication that they represent a genuine association. In fact, both proteins are regulatory subunits of the 26S proteasome. Therefore, our hypothesis is that protein pairs that are frequently co-purified in complexes are more likely to represent genuine associations than those that are not.

### Assessment of affinity-purified protein pairs

Accordingly, we produced sets of protein pairs from the affinity-purified complex dataset. A pair

Copyright © 2004 John Wiley & Sons, Ltd. 

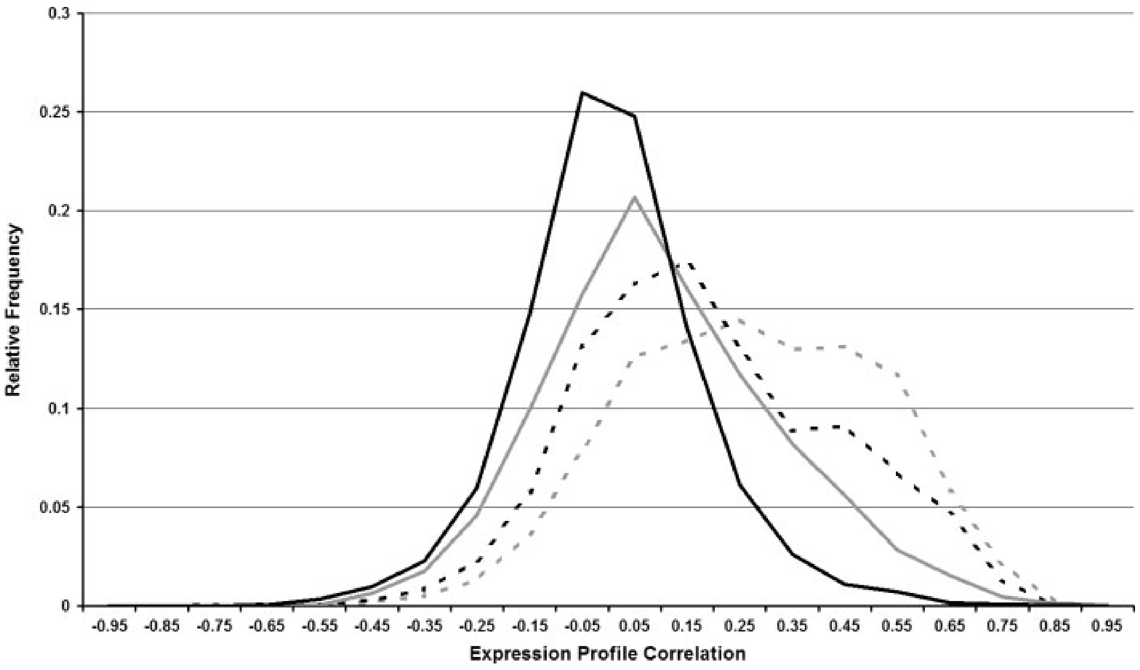

**Figure 10.** Frequency distribution of correlation of expression profiles for all frequently occurring pairs (FOPs) in affinity-purified protein complexes. Key: grey dashed line, HMS–PCI and TAP FOPs; black dashed line, TAP only FOPs; grey solid line, HMS–PCI only FOPs; black solid line, random pairs that occurs in more than one complex is defined as a frequently observed pair (FOP), while those that occur only once are defined as singly observed pairs (SOPs). Furthermore, those SOPs in which each protein occurs only once in that dataset are defined as unique SOPs (U-SOPs). For a list of all SOPs and FOPs, see the Supplementary Information.

We compared the composition of TAP and HMS–PCI complexes in terms of whether the proteins are involved in FOPs or SOPs. We found that 44% of the proteins in HMS–PCI complexes occur only in SOPs, compared to 21% in TAP. Also, only 3% of the proteins in HMS–PCI complexes were involved in U-SOPs, compared to 14% in TAP. Analysis of protein pairs using comparison of annotation (see Figure 9) shows that FOPs have higher 'match : no-match' ratios than SOPs. FOPs identified by both TAP and HMS–PCI have the highest scores, while those identified only by TAP have higher scores than those identified only by HMS–PCI. TAP U-SOPs have larger ratios than TAP SOPs. In contrast, HMS–PCI U-SOPs and SOPs give similar ratio values, which are not dissimilar to those generated by randomly chosen protein pairs. Analysis of expression profile correlation shows that FOPs tend to have much greater correlation coefficients than SOPs (see Figures 10 and 11). The correlation coefficients for SOPs are similar to those of random protein pairs. In addition, FOPs identified by both HMS–PCI and TAP have higher correlations than those chosen by only one method. These FOPs have a bimodal distribution, suggesting that there is a subset of highly correlated persistent associations.

It may be useful to distinguish such stable associations in protein interaction network maps, thus allowing a qualitative discrimination of the nature of the interactions. Figure 12 shows an interaction network containing protein pairs selected on the basis of each member of a pair being found in a TAP complex with the other member of the pair. The 10 proteins included in this map (Trs33p, Bet3p, Kre11p, Trs20p, Trs130p, Trs120p, Gsg1p, Bet5p, Trs31p and Trs23p) compose the entire TRAPP (Transport Protein Particle) complex, as listed in MIPS (MIPS currently lists 95 proteins involved in intracellular transport complexes, of which 10 are included in the TRAPP complex, category 260.60). Some of the

Copyright © 2004 John Wiley & Sons, Ltd. 

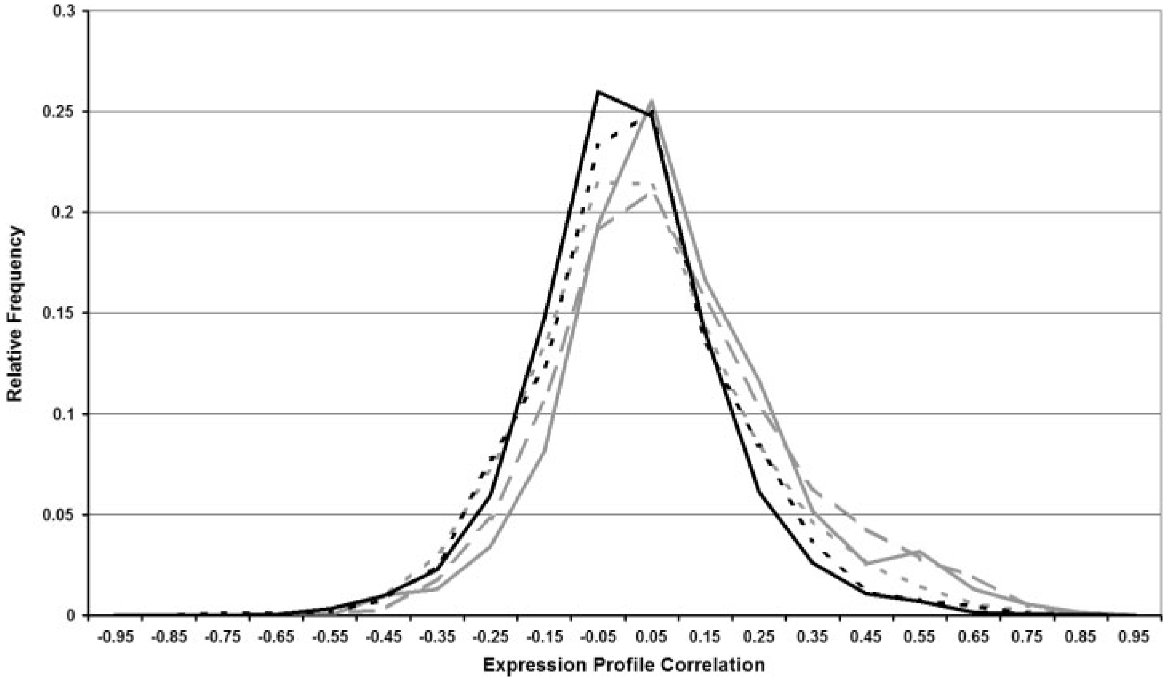

**Figure 11.** Frequency distribution of correlation of expression profiles for all single occurrence pairs (SOPs) in affinity-purified protein complexes. Key: grey dashed line, TAP SOPs; grey dotted line, HMS–PCI SOPs; grey solid line, TAP U-SOPs; black dotted line, HMS–PCI U-SOPs; black solid line, random pairs direct interactions (Trs20p–Bet3p, Trs31p–Bet3p, Trs20p–Trs31p, Trs31p–Bet5p) in this map have been determined by Y2H.

### Analysis of transiently-interacting proteins

One reason for differences between datasets could be that different experimental techniques are selecting different types of interactions. For example, HMS–PCI and Y2H might be better able to identify transient interactions than TAP (Aloy and Russell, 2002). This might be because HMS–PCI involves the overexpression of the bait protein and a single affinity purification step, while Y2H involves over-expression of both bait and identified proteins. Transient interactions are likely to have lower expression profile correlations than persistent interactions (Jansen *et al.*, 2002). Therefore, the fact that HMS–PCI protein pairs and Y2H interactions tend to have lower expression profile correlations than TAP protein pairs could be due these datasets containing a greater proportion of transient interacting proteins.

We generated a set of proteins likely to be associated in transient interactions and searched for these proteins in the Y2H, TAP and HMS–PCI datasets. We found 210 of these proteins present in the HMS–PCI complexes (out of a total of 1576 proteins) compared to 105 in the TAP complexes (out of a total of 1474 proteins). The occurrence of transiently interacting proteins in FOPs and SOPs was assessed: 9.2% of FOPs contained one or more of these proteins compared to 17.5% of SOPs. In Y2H interactions, we found 79 Set1 (17.0%) and 679 Set2 (14.3%) interactions contained transiently interacting proteins. These findings appear to support the assertion that transient interactions occur more frequently in Y2H and HMS–PCI data than in TAP data. In order to assess the effect of these interactions on our previous findings, we compared the expression profile correlations and annotation match : no-match ratios of FOPs and SOPs and Set1 and Set2 Y2H interactions which contained transiently interacting proteins.

The results (see Figures 13 and 14) show that the expression profile correlations of transient FOPs tend to be lower than non-transient FOPs, although still greater than transient SOPs. Similarly transient Set1 Y2H interactions tend to have smaller expression profile correlations than non-transient

Copyright © 2004 John Wiley & Sons, Ltd. 

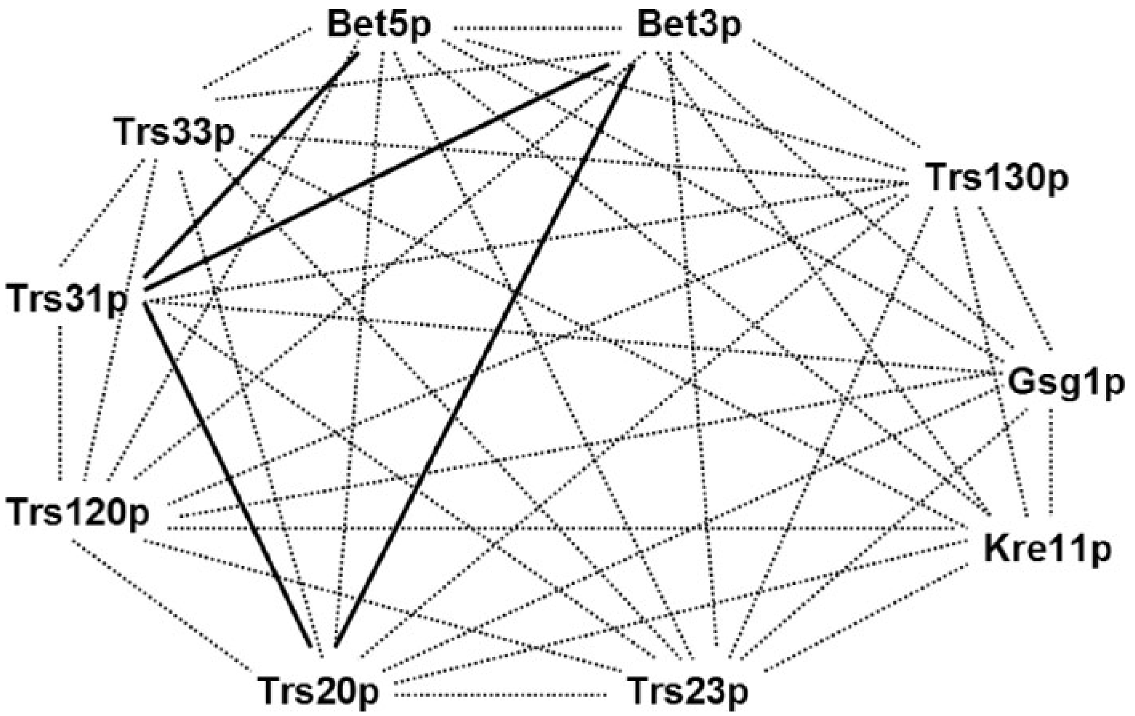

**Figure 12.** Protein association map showing FOP proteins found in TAP affinity-purified complexes. Solid lines show direct interactions shown by Y2H interactions, dashed lines indicate that pairs of proteins have been found in an affinity-purified complex. Note that Trs120p and TRS130p were identified by HMS–PCI using Gyp6 as bait. Trs31p–Bet3p, Trs31p–Trs20p and Trs20p–Bet3p interactions identified used both proteins as baits. Trs31p–Bet5p interaction was using Bet3p as bait. Other interactions involving these proteins have been identified by Y2H. These are: Cvt19p–Trs33p (by both Ito and Uetz using Trs33p as bait); Pho5–Bet3 (Pho5 as bait); Trs20–Yjr116p (Trs20 as bait); and Trs20–Srb2 (Srb2 as bait). Kre-11p has been used as a bait and identified Yor197p. Kre11p–Jsn1p was also identified using Jsn1 as bait. Gsg1p has been used as a bait and identified Yhb1p. Gsg1p–Yir040p was identified using Yir040p as bait. No Y2H interactions listed involving Trs130p, Trs120p and Trs23p

Set1 interactions. However, the annotation comparison results (Figures 15 and 16) indicate that transient FOPs have greater match : no-match ratios than transient SOPs and that transient Set1 Y2H interactions gave greater ratios than Set2 interactions. These results indicate that, while transiently interacting proteins may affect the frequency distribution of expression profile correlations, the differences in the annotation ratios remain. Therefore, transient FOPs appear more reliable than transient SOPs and transient Set1 interactions more reliable than transient Set2 interactions.

### Analysis of highly connected proteins

Protein networks generated from interaction data contain a subset of highly connected proteins (HCPs) that have a central role in linking together the numerous less-connected proteins (Jeong *et al.*, 2001). Our analysis of Y2H interactions indicates contamination by false-positives. To what extent does this contamination involve HCPs? To assess this, we generated interaction networks from all the Ito and Uetz Y2H data. The largest network produced contains 3170 proteins, involved in 4883 interactions. Interactions involving the 36 most highly connected proteins (those involved in more than 20 interactions) were assessed. The results (see Table 1) demonstrate some interesting trends.

First, very few HCP interactions have been verified by multiple datasets (i.e. are Set1 interactions). Of the 2161 interactions, only 67 (3.1%) occur in Set1. Set1 interactions make up 8.9% (466 out 5215) of the total interactions, so this value is significantly lower than expected by chance. Many HCPs have no interactions that are supported by multiple datasets. This includes Jsn1p, which is involved in 288 interactions. Of the 67

Copyright © 2004 John Wiley & Sons, Ltd. 

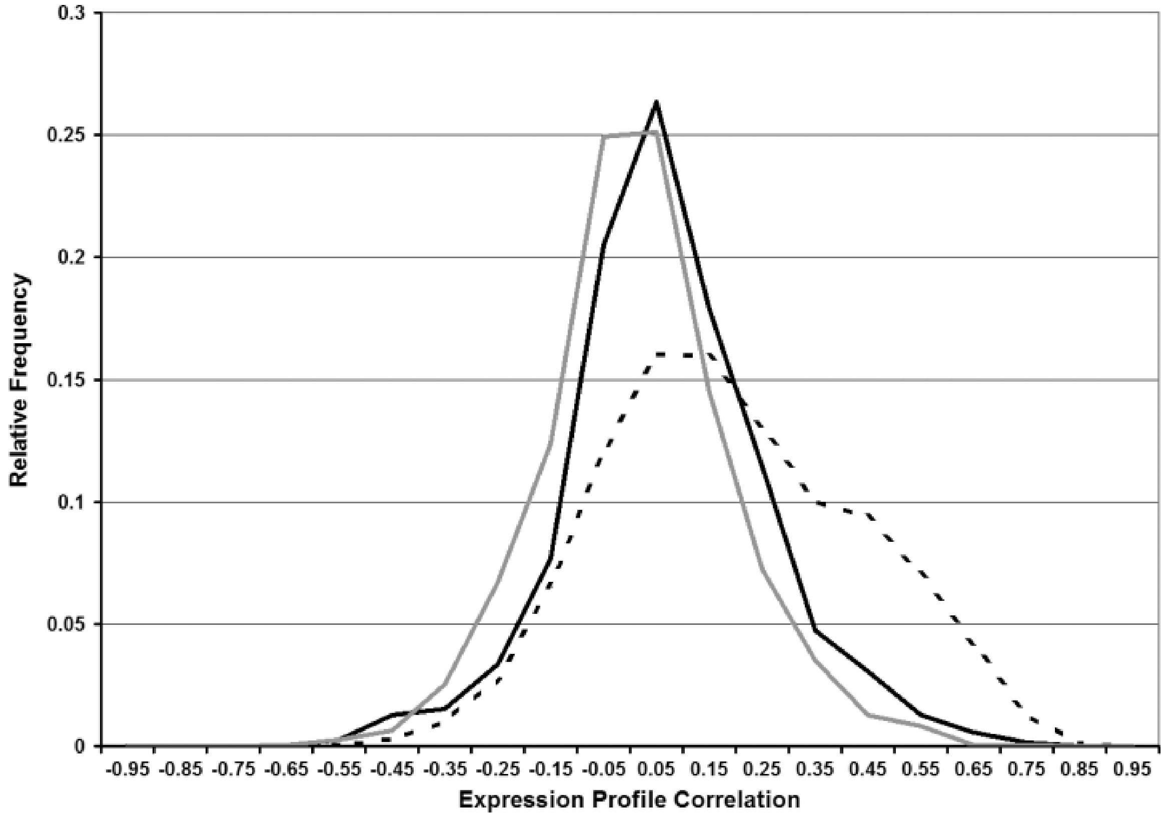

**Figure 13.** Frequency distribution of expression profile correlations for affinity-purified complex FOPs and SOPs containing transiently interacting proteins. Key: black solid line, FOPs containing transiently interacting protein; black dashed line, FOPs not containing transiently interacting proteins; grey solid line, SOPs containing transiently interacting proteins verified interactions, over half are associated with only three proteins, Apg17p (11 Set1 interactions), Srp1p (17 Set1 interactions) and Tem1p (seven interactions).

Second, in the vast majority of cases, unverified interactions were identified with the HCP as the bait protein. In only 221 of the 2161 interactions is the HCP the identified protein. Furthermore, 187 of these 221 interactions involve only nine HCPs. In only four of the 288 Y2H interactions involving Jsn1p is this protein the identified protein.

Third, when the identified protein is used as a bait, it rarely identifies the HCP. For example, of the 96 occasions on which a protein identified as interacting with Jsn1p was used as a bait protein, in no case was the reverse interaction identified.

### Discussion

Previous assessments of interaction datasets have also demonstrated contamination by false-positives and have allowed confidence scores to be associated with interactions. These assessments have included using gene expression (Kemmeren *et al.*, 2002), homology (Deane *et al.*, 2002) and network topology (Saito *et al.*, 2002; Goldberg and Roth, 2003).

However, it can be problematic to judge an individual interaction to be valid or a false-positive on the basis of other data. For example, correlation of mRNA expression is not necessary for protein interaction (Bader *et al.*, 2004) and highly correlated expression does not necessarily indicate a genuine interaction. This might explain the different annotation match : no-match ratios observed for Kemmeren Set1 and Set2 interactions. The methodology we have chosen for analysing interactions does not allow us to give an estimate of the reliability of a particular interaction. Instead we have produced sets of interactions on the basis of overlapping datasets and testing the assumption that these sets are of equal quality. Our results

Copyright © 2004 John Wiley & Sons, Ltd.

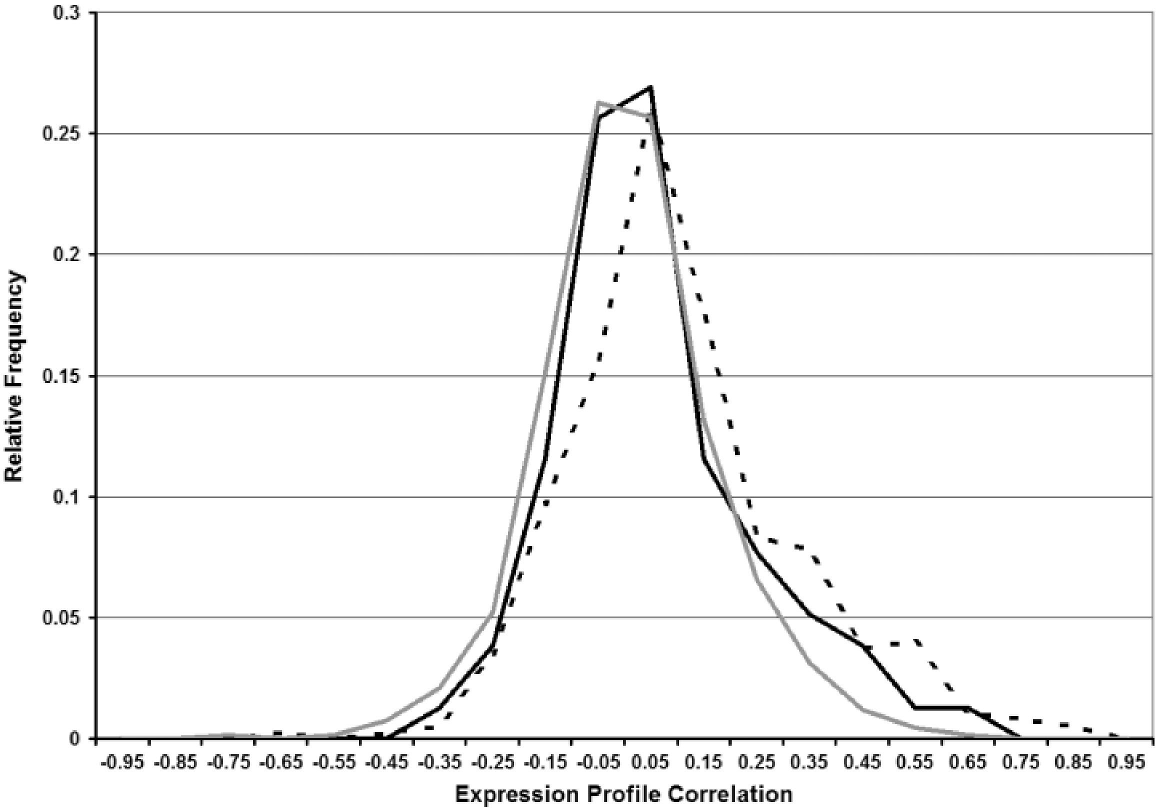

**Figure 14.** Frequency distribution of expression profile correlations for Set1 and Set2 Y2H interactions containing transiently interacting proteins. Key: black solid line, Set1 interactions containing transiently interacting proteins; black dashed line, Set1 interactions not containing transiently interacting proteins; grey solid line, Set2 interactions containing transiently interacting proteins demonstrate that interactions supported by multiple datasets perform much better than those supported by single datasets, and appear more likely to be genuine interactions. Analysis of expression profile correlation indicates that, while high correlation values may not be a requirement for protein interaction, those interactions supported by multiple datasets tend to be more positively correlated than those occurring in a single dataset. Annotation match : no-match ratios show a consistent trend for each of the four annotations (i.e. interactions supported by multiple datasets have higher ratios). However, the number of matches obtained for GO annotations is always lower than those obtained from MIPS annotations. This suggests that the GO terms are more specific and, therefore, more difficult to match exactly.

Because we do not consider individual interactions, it is difficult to give an exact number of reliable interactions in the datasets. However, the observed differences between Set1 and Set2 interactions suggest a lower figure than the 50% previously predicted (Deane *et al.*, 2002).

It is also clear that some datasets perform better than others, TAP-purified complexes have higher annotation comparison ratios and expression profile correlations than those purified using HMS–PCI, while Uetz Y2H interactions score better than Ito interactions (although the Ito core dataset produce similar scores to Uetz). There is evidence that differences in the experimental methods used for affinity purification have resulted in differences in the characteristics of the resulting complexes. We have found evidence to support this, based on our selection of proteins likely to be involved in transient interactions. However, while transient interactions may result in lower expression profile correlations, they do not appear to be responsible for the differences in match : no-match ratios between TAP, HMS–PCI and Y2H. Moreover, increased

Copyright © 2004 John Wiley & Sons, Ltd. 

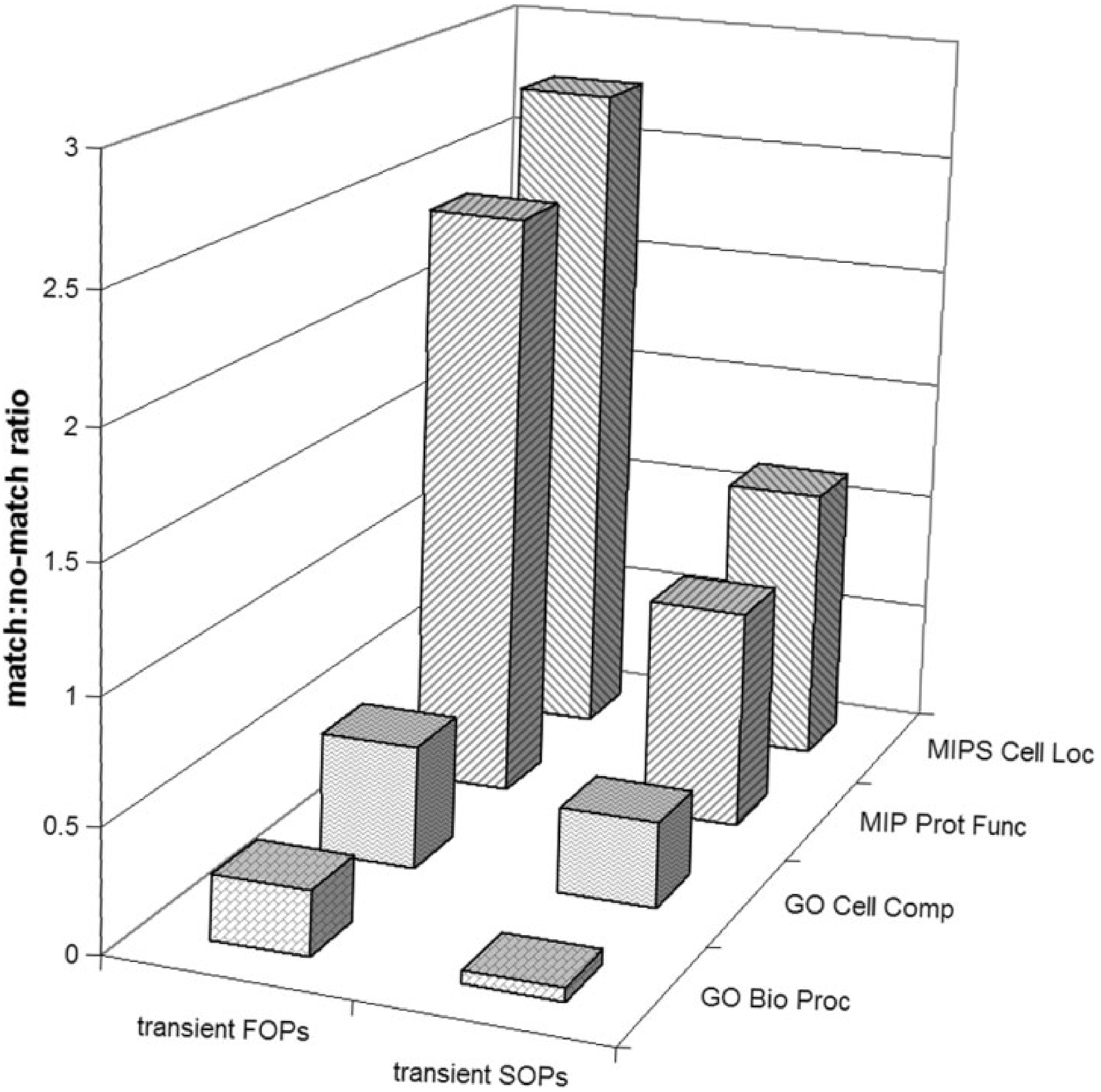

**Figure 15.** Annotation match : no-match ratios for affinity-purified complex FOPs and SOPs containing transiently interacting proteins sensitivity to transient interactions does not explain the lack overlap between TAP and HMS–PCI complexes purified using the same bait. If increased numbers of transient interactions were the only reason for the differences between HMS–PCI and TAP datasets, we would expect that HMS–PCI complexes would contain all TAP complexes plus a set of transiently interacting proteins.

Clearly, large-scale contamination of interactome data by false-positives has serious implications. Interaction data has been used to generate networks and has shown the high connectivity of essential proteins (Jeong *et al*., 2001). However, as we have demonstrated, these proteins are likely to be associated with large numbers of false-positive interactions. This might explain why highly connected proteins are not subject to more evolutionary constraints (Wagner, 2002; Hahn *et al*., 2004).

Interaction data have been used to assign protein function to previously unannotated proteins (e.g. Deng *et al*., 2002; Pereira-Leal *et al*., 2003; Samanta and Liang, 2003). Clearly, the accuracy

Copyright © 2004 John Wiley & Sons, Ltd. 

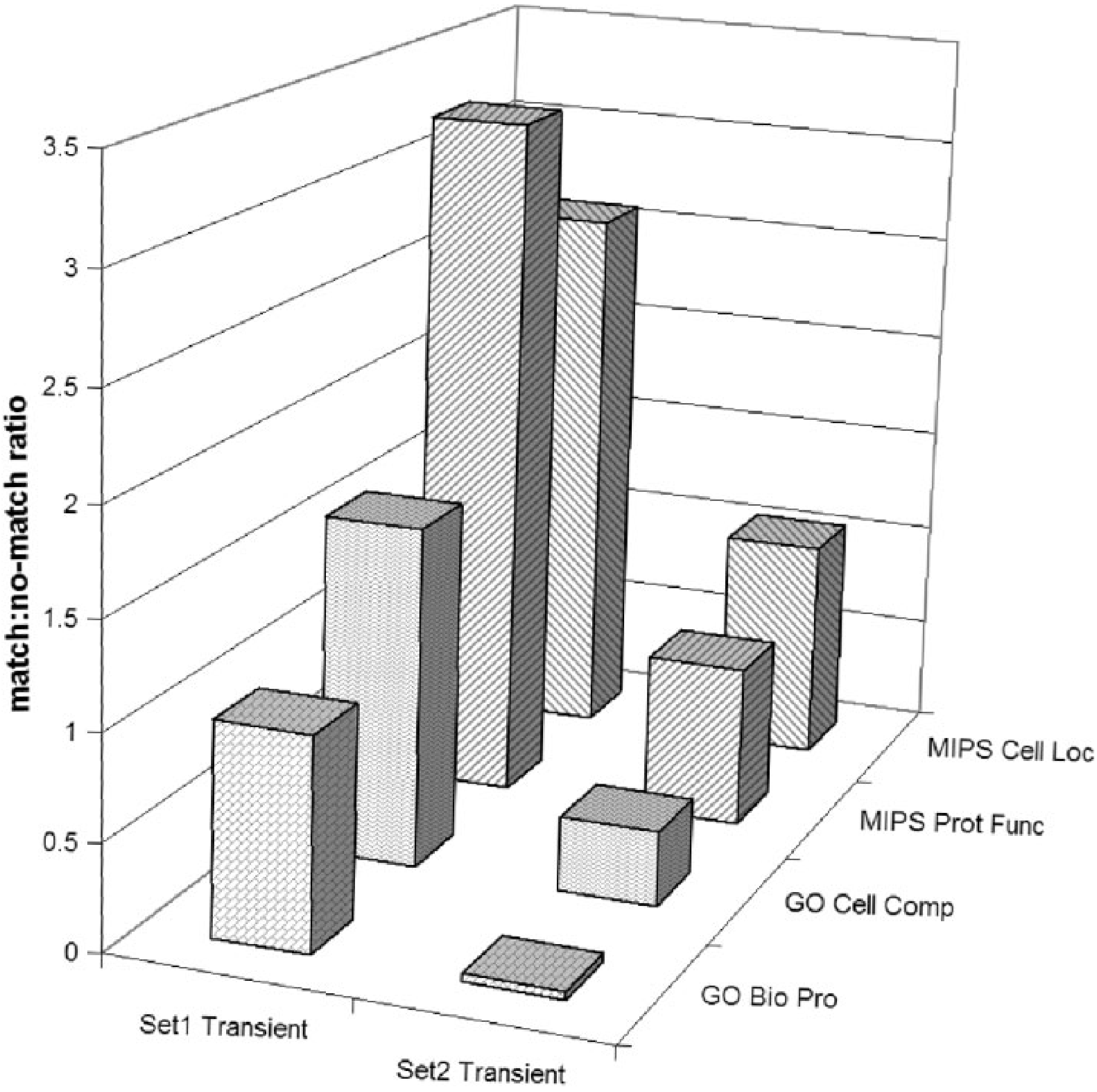

**Figure 16.** Annotation match : no-match rations for Set1 and Set2 Y2H interactions containing transiently interacting proteins of function predictions will be influenced by the accuracy of the interaction data on which the predictions are based. Often poor data quality has been acknowledged in the data analysis. For example, an analysis of functional topology in an interaction network (Przulj *et al.*, 2004) restricts analysis to the top 11 000 interactions from von Mering *et al.* (2002), rather than the total 78 000 interactions in the yeast interactome, while an assessment of the yeast interactome size (Grigoriev, 2003) excludes the highly connected proteins Tem1p, Srp1p and Jsn1p. We have demonstrated that integrating protein–protein interaction data with functional datasets allows users to extract reliable sets of interactions. We make these integrated data available to the research community both in the Supplementary Information to this paper and via the GIMS data warehouse itself (Cornell *et al.*, 2003), which also provides tools with which readers may perform their own analyses.

Copyright © 2004 John Wiley & Sons, Ltd. 

**Table 1.** Analysis of interactions involving highly connected protein (HCP) Interactions

| HCP | Interactions | Set1 interactions | HCP is identified protein | HCP is bait protein | Id protein used as bait |
|---|---|---|---|---|---|
| YGL181W(GTS1) | 23 | 0 | 4 | 19 | 6 |
| YPL070W | 30 | 1 | 2 | 27 | 20 |
| YOR128C(ADE2) | 23 | 0 | 20 | 3 | 1 |
| YLR347C(KAP95) | 27 | 0 | 0 | 27 | 19 |
| YDL153C(SAS10) | 32 | 0 | 0 | 32 | 10 |
| YGL070C(RPB9) | 44 | 0 | 0 | 44 | 12 |
| YJR091C(JSN1) | 288 | 0 | 4 | 284 | 96 |
| YDR259C(YAP6) | 23 | 1 | 20 | 2 | 1 |
| YLR291C(GCD7) | 23 | 3 | 1 | 19 | 15 |
| YDL239C(ADY3) | 31 | 2 | 5 | 24 | 14 |
| YML064C(TEM1) | 72 | 7 | 0 | 65 | 49 |
| YLR453C(RIF2) | 78 | 0 | 1 | 77 | 28 |
| YOR047C(STD1) | 31 | 1 | 26 | 4 | 1 |
| YKL002W(DID4) | 41 | 1 | 2 | 38 | 14 |
| YOR264W | 26 | 1 | 0 | 25 | 7 |
| YLR295C(ATP14) | 124 | 0 | 1 | 123 | 43 |
| YPR086W(SUA7) | 98 | 0 | 0 | 98 | 29 |
| YLR423C(APG17) | 62 | 11 | 42 | 9 | 5 |
| YGR218W(CRM1) | 34 | 0 | 1 | 33 | 11 |
| YER022W(SRB4) | 98 | 5 | 3 | 90 | 28 |
| YDR510W(SMT3) | 22 | 2 | 20 | 0 | 0 |
| YLR373C(VID22) | 31 | 0 | 0 | 31 | 10 |
| YER081W(SER3) | 95 | 1 | 1 | 93 | 28 |
| YNL092W | 29 | 0 | 3 | 26 | 9 |
| YLR288C(MEC3) | 79 | 2 | 3 | 74 | 22 |
| YGL127C(SOH1) | 69 | 0 | 2 | 67 | 31 |
| YDL100C | 25 | 1 | 21 | 3 | 1 |
| YIR038C(GTT1) | 21 | 0 | 21 | 0 | 0 |
| YHR114W(BZZ1) | 91 | 0 | 0 | 91 | 36 |
| YDR311W(TFB1) | 24 | 4 | 0 | 20 | 9 |
| YMR153W(NUP53) | 25 | 2 | 17 | 6 | 3 |
| YLR447C(VMA6) | 88 | 2 | 0 | 86 | 35 |
| YDR318W(MCM21) | 34 | 0 | 1 | 33 | 6 |
| YNL189W(SRP1) | 132 | 17 | 0 | 115 | 61 |
| YDR034C(LYS14) | 63 | 0 | 0 | 63 | 21 |
| YMR047C(NUP116) | 125 | 3 | 0 | 122 | 47 |

Key: Interactions, number of Y2H interactions involving HCP; Set1 interactions, number of these interactions which are in Set1; HCP is identified protein, number of interactions not in Set1 in which the HCP is not the bait protein; HCP is bait protein, number of interactions not in Set1 in which the HCP is the bait protein; Id protein used as bait, number of proteins identified by HCP which have been used as a Y2H bait protein but have not identified the HCP (i.e. the reverse interaction has been tested but not found).

### References

Aloy P, Russell RB. 2002. The third dimension for protein interactions and complexes. *Trends Biochem Sci* **27**: 633–638.

Ashburner M, Ball CA, Blake JA, *et al*. 2000. Gene ontology: tool for the unification of biology. *Nature Genet* **25**: 25–29.

Bader GD, Hogue CW. 2002. Analyzing yeast protein–protein interaction data obtained from different sources. *Nature Biotechnol* **20**: 991–997.

Bader JS, Chaudhuri A, Rothberg JM, Chant J. 2004. Gaining confidence in high-throughput protein interaction networks. *Nature Biotechnol* **22**: 78–85.

Blackstock W, Mann M. 2001. A boundless future for proteomics? *Trends Biotechnol* **19**: S1–S2.

Cornell M, Paton NW, Hedeler C, *et al*. 2003. GIMS: an integrated data storage and analysis environment for genomic and functional data. *Yeast* **20**: 1291–1306.

Deane CM, Salwinski L, Xenarios I, Eisenberg D. 2002. Protein interactions: two methods for assessment of the reliability of high throughput observations. *Mol Cell Proteomics* **1**: 349–356.

Deng M, Zhang K, Mehta S, Chen T, Sun F. 2003. Prediction of protein function using protein–protein interaction data. *J Comput Biol* **10**: 947–960.

Gavin AC, Boesche M, Krause R, *et al*. 2002. Functional organization of the yeast proteome by systematic analysis of protein complexes. *Nature* **415**: 141–147.

Copyright © 2004 John Wiley & Sons, Ltd. 

Gu W, Malik S, Ito M, *et al*. 1999. A novel human srb/med-containing cofactor complex, smcc, involved in transcription regulation. *Mol Cell* **3**: 97–108.

Goldberg DS, Roth FP. 2003. Assessing experimentally derived interactions in a small world. *Proc Natl Acad Sci USA* **100**: 4372–4376.

Grigoriev A. 2003. On the number of protein–protein interactions in the yeast proteome. *Nucleic Acids Res* **31**: 4157–4161.

Hahn MW, Conant GC, Wagner A. 2004. Molecular evolution in large genetic networks: does connectivity equal constraint? *J Mol Evol* **58**: 203–211.

Hazbun TR, Fields S. 2001. Networking proteins in yeast. *Proc Natl Acad Sci USA* **98**: 4277–4278.

Ho Y, Gruhler A, Heilbut A, *et al*. 2002. Systematic identification of protein complexes in *Saccharomyces cerevisiae* by mass spectrometry. *Nature* **415**: 180–183.

Hughes TR, Marton MJ, Jones AR, *et al*. 2000. Functional discovery via a compendium of expression profiles. *Cell* **102**: 109–126.

Ito T, Chiba T, Ozawa R, *et al*. 2001. A comprehensive two-hybrid analysis to explore the yeast protein interactome. *Proc Natl Acad Sci USA* **98**: 4569–4574.

Jansen R, Greenbaum D, Gerstein, M. 2002. Relating whole-genome expression data with protein–protein interactions. *Genome Res* **12**: 37–46.

Jeong H, Mason SP, Barabasi A-L, Oltvai ZN. 2001. Lethality and centrality in protein networks. *Nature* **411**: 41–42.

Kemmeren P, van Berkum NL, Vilo J, *et al*. 2002. Protein interaction verification and functional annotation by integrated analysis of genome-scale data. *Mol Cell* **9**: 1133–1143.

Mewes HW, Albermann K, Heumann K, Liebl S, Pfeiffer F. 1997. MIPS: a database for protein sequences, homology data and yeast genome information. *Nucleic Acids Res* **25**: 28–30.

Paton NW, Khan SA, Hayes A, *et al*. 2000. Conceptual modelling of genomic information. *Bioinformatics* **16**: 548–557.

Pereira-Leal JB, Enright AJ, Ouzounis CA. 2004. Detection of functional modules from protein interaction networks. *Proteins* **54**: 49–57.

Przulj N, Wigle DA, Jurisica I. 2004. Functional topology in a network of protein interactions. *Bioinformatics* **20**: 340–348.

Rain JC, Selig L, De Reuse H, *et al*. 2000. The protein–protein interaction map of *Helicobacter pylori*. *Nature* **409**: 211–215.

Saito R, Suzuki H, Hayashizaki Y. 2002. Interaction generality, a measurement to assess the reliability of a protein–protein interaction. *Nucleic Acids Res* **30**: 1163–1168.

Samanta MP, Liang S. 2003. Predicting protein functions from redundancies in large-scale protein interaction networks. *Proc Natl Acad Sci USA* **100**: 12 579–12 583.

Uetz P, Giot L, Cagney G, *et al*. 2000. A comprehensive analysis of protein–protein interactions in *Saccharomyces cerevisiae*. *Nature* **403**: 623–627.

von Mering C, Krause R, Snel B, *et al*. 2002. Comparative assessment of large-scale datasets of protein–protein interactions. *Nature* **417**: 399–403.

Copyright © 2004 John Wiley & Sons, Ltd. 
